# Resistance to β-Lactams in *Neisseria ssp* Due to Chromosomally Encoded Penicillin-Binding Proteins

**DOI:** 10.3390/antibiotics5040035

**Published:** 2016-09-28

**Authors:** André Zapun, Cécile Morlot, Muhamed-Kheir Taha

**Affiliations:** 1 Institut de Biologie Structurale (IBS), University Grenoble Alpes, CEA, CNRS, Grenoble 38044, France; cecile.morlot@ibs.fr; 2 Invasive Bacterial Infection and National Reference Center for Meningococci, Institut Pasteur, Paris 75015, France; muhamed-kheir.taha@pasteur.fr

**Keywords:** *Neisseria meningitidis*, *Neisseria gonorrhoeae*, β-lactam resistance, penicillin-binding proteins

## Abstract

*Neisseria meningitidis* and *Neisseria gonorrhoeae* are human pathogens that cause a variety of life-threatening systemic and local infections, such as meningitis or gonorrhoea. The treatment of such infection is becoming more difficult due to antibiotic resistance. The focus of this review is on the mechanism of reduced susceptibility to penicillin and other β-lactams due to the modification of chromosomally encoded penicillin-binding proteins (PBP), in particular PBP2 encoded by the *penA* gene. The variety of *penA* alleles and resulting variant PBP2 enzymes is described and the important amino acid substitutions are presented and discussed in a structural context.

*Neisseria meningitidis* and *Neisseria gonorrhoeae* are pathogens that have acquired reduced susceptibility to penicillin and other β-lactams via two routes. The modification of at least one chromosomally encoded penicillin-binding protein is the focus of this review. Alternatively, resistance to β-lactams may result from the production of a plasmid-encoded β-lactamase, which is common and also clinically relevant in *N. gonorrhoeae* [1,2,3], while it is rare in *N. meningitidis* [4]. Mutation in a porin and over expression of an efflux pump were also shown to contribute in *Neisseria gonorrhoeae* to β-lactam resistance [5].

Penicillin-binding proteins are the enzymes that assemble the peptidoglycan on the outer side of the plasma membrane to constitute the cell wall. Glycan chains are polymerized by a transglycosylase activity, whereas peptides attached to the saccharide units are cross-linked by a transpeptidase activity. The transpeptidase activity is normally inhibited by β-lactams that form a covalent adduct to the active site serine residue, owing to their high reactivity and likeness to the terminal dipeptide that is the physiological substrates for the transpeptidation [6,7]. *Neisseria* species have four PBPs. PBP1 is a bifunctional transglycosylase and transpeptidase, PBP2 is a monofunctional transpeptidase, whereas PBP3 and PBP4 are low-molecular weight carboxypeptidases/endopeptidases [8]. Note that PBP4 is not revealed by labeling with radio-active β-lactams in vivo, although it is highly reactive in vitro. PBP4 is likely not expressed at high level in laboratory conditions.

*N. gonorrhoeae* and *N. meningitidis* are exclusively human bacteria and are the two pathogenic species of the genus *Neisseria*. Both bacteria interact with mucosal surfaces (mainly the nasopharynx for meningococci and the genito-urinary tract for gonococci). Meningococci may cross the epithelial barrier to invade and spread through the bloodstream, often causing fatal *purpura fulminans*, to reach the central nervous system, resulting in meningitis. Gonococci colonize and invade the epithelium of the genito-urinary tract and provoke local infection (gonorrhea) but rarely invade blood to cause systemic infections. The presence of gonococci, frequently asymptomatic, in the rectum and/or in the pharynx (rectal and pharyngeal gonorrhea) is mostly described in men who have sex with men. Moreover, meningococcal urethritis is also described in men and frequently among men who have sex with men [9]. The possibility that meningococci and gonococci share the same niche may allow gene transfer (including those involved in resistance to antibiotics) between these two species. Indeed, frequent horizontal DNA exchanges occur among species of the genus *Neisseria* as discussed below [10].

*N. meningitidis* is encapsulated and 12 capsular types (called serogroups) are currently described. However, six of these recognized serogroups (A, B, C, W, X, and Y) are responsible for almost all cases of invasive meningococcal disease (IMD) but with variable distribution worldwide [11]. Invasive meningococcal infections are mandatory reportable diseases in many countries and are considered epidemic prone by the World Health Organisation (WHO). Meningococci cause more than 1.2 million cases of IMD and 135,000 deaths each year across the globe [12], with important regional differences. The incidence of IMD in Europe and North America, is low (around 1 per 100,000 people) and the disease is endemic and characterized by seasonal peaks and small clusters of cases [13]. At the opposite, the incidence in the “meningitis belt” of sub-Saharan Africa is much higher, with large periodic epidemics that occur frequently with an incidence that may reach 1000 per 100,000 people [12].

The WHO estimates that there were more than 100 million new cases of gonorrhea among adults worldwide in 2008. The number of cases is increasing, in particular in the Western Pacific, South-East Asia and Africa [14]. Moreover, gonococcal strains showing multidrug resistance to antibiotics are increasingly described [15].

In the late 70s, gonococcal strains with reduced susceptibility to β-lactams that do not express a β-lactamase were found to exhibit reduced labeling of PBP2 and PBP1 with radiolabeled penicillin [16]. Reduced labeling of PBP2 was later observed in meningococci [17]. Strains of *N. meningitidis* with PBP2 showing a reduced affinity to penicillin G were found to have altered peptidoglycan structure, with an accumulation of pentapeptides suggesting a lower tranpeptidase or carboxypeptidase activity [18].

PBP2 is encoded by the *penA* gene. A major European study determined a partial nucleotide sequence of *penA* from 1670 meningococcal strains isolated over 6 decades. Hundred thirty-nine alleles were uncovered, including 38 very similar sequences from susceptible strains, and 101 highly diverse sequences from strains with a diminished susceptibility to penicillin [19]. A Swedish study revealed similar findings [20]. The total current (15 July 2016) number of different *penA* alleles at the nucleotide level is 617, which translates into 137 different PBP2 proteins in meningococci. At the amino acid level in *N. gonorrhoeae*, over 50 different full sequences have been reported which are commonly ascribed a Roman numeral [21,22,23,24,25,26,27,28,29] (Figure 1). Although no global clonal expansion was detected, some clones can be highly successful locally. For example, in a clinic for sexually transmitted diseases in the Netherlands, the single sequence XXXIV was identified in 53 of 128 cefotaxime-resistant isolates [30].

*Neisseria* species are naturally competent organisms, and horizontal gene transfers are common [40]. The mechanism of acquisition of non-plasmidic resistance in *Neisseria* is therefore similar to that of *Streptococcus pneumoniae* [41,42]. Multiple events of homologous recombination between related sequences can give rise to mosaic genes, and *penA* is often mosaic in resistant strains of *N. gonorrhoeae* [31] and *N. meningitidis* [43]. Indeed, horizontal transfer of a *penA* allele conferring resistance was observed during co-cultivation of drug-susceptible and resistant gonococcal strains [44]. Additional point mutations were found to arise during this experiment [44].

The origin of the foreign sequence fragments that are found in the *penA* mosaic genes of clinical resistant gonococci and meningococci has been investigated in some depth. Several commensal species, such as *Neisseria flavescens*, *Neisseria cinerea*, or *Neisseria perflava*, appear to have each contributed sequence blocks to *penA* genes from resistant gonococcal strains [43,45,46,47]. In *N. meningitidis*, an analysis of a 402 bp-fragment of the *penA14* allele encoding the C-terminus of PBP2 found contributions from *N. flavescens*, *N. cinerea*, *N. mucosa* and *N. perflava* [19]. *N. flavescens* isolates recovered from the pre-antibiotic era have relatively high penicillin minimal inhibitory concentrations and a PBP2 with an intrinsic low affinity for penicillin [45]. Transfer in the laboratory of the *penA* gene from such *N. flavescens* isolates could indeed confer some resistance to *N. meningitidis* [45]. In contrast, *N. cinerea* is not naturally resistant, and accordingly, no resistance was achieved in *N. meningitidis* upon transfer of the *penA* gene from this species [45]. In some publications, PBP2 sequences with few substitutions such as sequence XIII from *N. gonorrhoeae* have been described as non-mosaic, by contrast with sequences that have many substitutions (such as sequence X) [48]. However, nucleotide sequences alignments reveal that the short modified segment spanning residue 504 to 516 in sequence XIII may have originated from *N. perflava*. Also, the five substitutions F504L, A510V, I515V, H541N and I566V, that collectively confer resistance to *N. meningitidis*, were also found (all or the three latter) in nearly half of 128 carriage isolates of *Neisseria lactamica* [49].

PBP2 sequences of *N. cinerea* origin found in resistant meningococci have an additional aspartic acid following D346 (D346a), which is not present in susceptible *N. cinerea* strains [45]. This insertion was also found in PBP2 sequences from many resistant gonococcal strains (Figure 1). Site-directed mutagenesis has demonstrated that this insertion is sufficient to decrease the reactivity of PBP2 for β-lactams and to confer some resistance to *N. gonorrhoeae* [32]. A clinical resistant strain was later discovered that only has this additional aspartic acid [21]. The consequences of this insertion have been investigated in some depth (see below).

Several crystal structures of gonococcal PBP2 have now been solved and provide molecular details for the mechanism of resistance. PBP2 from a penicillin-susceptible strain was solved to a resolution of 2.4 Å [35]. More recently, the penicillin-binding domain of PBP2 with sequence XII (XXXVI), which carries four substitutions (F504L, A510V, A516G and P551S) in addition to the insertion D346a, has been solved at a resolution of 2.2 Å [33] (Figure 2A). Previously, the structure of PBP2 with the same sequence without the D346a insertion (F504L, A510V, A516G and P551S) had been reported [35].

Important features of the PBP structure can be summarized as follows. PBPs are characterized by the presence of a penicillin-binding domain (Figure 2), which harbors three specific motifs: SXXK, (S/Y)XN and (K/H)(S/T)G. The penicillin-binding domain is characterized by an active site cleft between an α-helical sub-domain and an α/β-sub-domain, which consists of a 5-stranded β-sheet covered by a C-terminal α-helix. The first motif SXXK is on the N-terminus of helix α2 of the helical sub-domain, on the bottom left of the active site groove, in the orientation shown in Figure 2. The hydroxyl of the serine of the SXXK motif is the active site group that undergoes attack on the peptide bond of the dAla-dAla dipeptide of the peptidoglycan or to β-lactam ring of the drugs. The third KTG motif on strand β3 of the α/β sub-domain is located on the bottom right of the active site. The second SXN motif is on the top side of the active site, on a loop between helix 4 and 5 of the helical sub-domain.

The acylation of PBPs by β-lactams comprises two steps. The first step is the formation of a preacylation non-covalent complex characterized by a dissociation constant *K*_D_. The second step is the opening of the β-lactam ring following the attack of the hydroxyl of the active site serine onto the carbonyl of the drug, resulting in the covalent attachment of the antibiotic. The second step is characterized by the first order rate constant *k*_2_. The rate of the overall reaction of acylation is determined for any drug concentration by the second order rate constant *k*_2_/*K*_D_, which is termed the acylation efficiency. In PBP2 XII (which harbors one insertion and 4 substitutions), the efficiency of acylation by penicillin or bocillin (a fluorescent derivative of penicillin) is diminished about 15-fold [33,35]. PBP2s with only the D346a insertion, or conversely only the four F504L, A510V, A516G and P551S substitutions have their acylation efficiencies diminished only 5-fold [33,35].

In gonococcal PBP2, D346 is involved in a hydrogen-bond network with the second catalytic motif SSN (361–363). The structure of PBP2 XII revealed that very little is changed in the active site. D346 retains the same interactions as in the susceptible sequence. The structural comparison of different PBP structures has emphasized the importance of the hydrogen-bonding of the second catalytic motif [50]. Indeed, the middle serine of the SSN motif of pneumococcal PBP2x is also hydrogen-bonded to an aspartate similarly located as gonococcal D346 on the β2a-β2d hair-pin loop. In PBP2a from *S. aureus*, the middle residue of the SXN motif is an asparate that makes a salt bridge to a lysine in the β2a-β2d hair-pin loop. In *Mycobacterium tuberculosis* PBPA, this linkage is provided by a covalent disulfide bond [50]. It was therefore proposed that the additional acidic residue likely interferes with this hydrogen-bonding network, causing a five-fold decrease in the acylation efficiency [50]. Alternatively, the structure of PBP2 XII also suggests that the additional Asp residue, which is positioned on the C-side of the native Asp346, projects in the active site and likely interferes with the antibiotic binding (Figure 3). The formation of the preacylation complex may be hindered by the presence of the negatively charged side chain of D346a in a position that would normally accept the hydrophobic R1 substituent of penicillin [33]. Note that the two explanations are not mutually exclusive. Surprisingly, only an additional Asp was tolerated in vivo and maintained viability; no other residues were tolerated.

The structure of PBP2 XII without the D346a insertion, a sequence not found in the clinical strain, showed very little structural modification, although the four substitutions in positions 504, 510, 516 and 551 also caused a five-fold reduction of the acylation efficiency, mostly due to the F504L and P551S substitutions, and a drop in thermal stability [35]. Note that residues 504 and 510 are in the β3–β4 loop. Flexibility of this loop has been linked to β-lactam resistance in PBP2x from *S. pneumoniae* [51].

The substitutions at position 501 in gonococcal sequences is another example of a point mutation that arose in addition to mosaicism. Indeed, the A501V or A501P substitutions are absent from related species that contributed sequence fragments to resistant *N. gonorrhoeae* [52], but were found in sequences of clinical origin. The A501V mutation was found to be somewhat correlated with strains expressing resistance to ceftriaxone, a third generation cephalosporin [23]. This was confirmed experimentally, as introduction of the A501V mutation in a mosaic PBP2 (XXVIII) normally devoid of this substitution increased the resistance to ceftriaxone and cefixime (cephalosporins) while decreasing the resistance to penicillin [34]. The A501V substitution in sequence II that harbors the D436a insertion, also increases 3-fold resistance to cephalosporins [36]. The spontaneous A501V substitution in sequence XII was also selected in the laboratory on ceftriaxone resulting in sequence XIII, thus recapitulating an event that had occurred in nature [37]. The effect was mirrored in vitro on the acylation efficiency *k*_2_/*K*_D_ with cefixime and penicillin, in that introduction of the A501V substitution increased the reactivity with penicillin and decreased that with cefixime. Position 501 is close to the active site Ser310 at the beginning of the loop connecting strands β3 and β4 which is disordered in the crystal structure of PBP2 [35] (Figure 2B).

An A501P substitution is found in the mosaic sequence XXXIX from a strain that showed high resistance to third-generation cephalosporins [38]. Sequence XXXIX is otherwise identical to PBP2 XXXIV. Introduction of either sequences in the same strain has shown that the A501P substitution increases resistance to cephalosporins up to 30-fold but resistance to carbapenems was abolished [36]. Penicillins, cephalosporins and carbapenems differ by the nature of the ring fused to the core β-lactam ring (thiazolidine, 3,6-dihydro-2H-1,3-thiazine, and 2,3-dihydro-1H-pyrrole, respectively). The substitution at position 501 highlights the fact that subtle differences in the active site can discriminate between small modifications of the drug.

It is usually difficult to pinpoint the substitutions that contribute to the diminished reactivity of altered PBPs for β-lactams, due to the process of homologous recombination that swaps large fragments to generate the mosaic genes. However, several studies have uncovered substitutions that contribute particularly to the emergence of the gonococcal resistance to third-generation cephalosporins.

In studies of PBP2, recombination of partial sequences of *penA* from clinical strains with intermediate resistance to cefixime allowed us to identify a subset of substitutions that contribute to resistance [22,34]. Further narrowing by site-directed mutagenesis pointed out I312M, V316T and G545S as contributing most to the reduction of reactivity towards most cephems. The I312M substitution takes place within the SAIK first catalytic motif, and is therefore analogous to the M339F substitution characterized in *S. pneumoniae* PBP2x [53]. The V316T substitution is in the middle of helix α2 which starts with the active site S310. V316 is one turn downstream of K313 from the SAIK catalytic motif, and their side chains protrude on the same side of α2. It is therefore likely that the V316T substitution impacts the spatial arrangement of the active site. G545 sits at the beginning of α11 facing strand β3, which lines the active site. The introduction of a serine side chain at position 545 likely modifies the conformation of β3 and the active site. These effects are certainly subtle since they appear to differently affect the resistance to various cephems [22,34]. These three mutations display epistasis with other substitutions present in the mosaic PBP2 from which they were identified, in that their introduction in a wild-type strain confers only modest resistance, whereas their reversion in the originating mosaic PBP2 abolishes resistance [34].

N512Y is another substitution that was found to contribute to the resistance to cephalosporin [34]. Reversion of N512Y greatly diminished resistance to ceftriaxone and cefixime, while not affecting resistance to penicillin. Residue 512 is in the loop connecting β3 and β4 like residues 504 and 510. However, reversion of F504L or A510V had little effect on resistance to cephems but diminished the resistance to penicillin [34]. The physiological results of the mutations were broadly in agreement with the measured kinetics of the reaction between recombinant enzymes and β-lactams.

The surveillance and characterization of gonococcal strains has permitted the identification of novel substitutions in sequence F2Z7K9 from a Japanese strain with high resistance to ceftriaxone. This sequence is similar to sequence XXVIII from strains with intermediate resistance to cephems, with 13 differences, three of which were shown to be very important [54]. Within the SXXK catalytic motif, the A311V substitution is found in addition to the I312M substitution already present in sequence XXVIII. The substitution V316T of sequence XXVIII is replaced by a V316P substitution. Finally, the T483S substitution has a great influence on the reactivity with β-lactams. Residue 483 is in a loop facing strand β3, which carries the KTG motif, and protrudes into the active site. These three substitutions have similar effects on the reactivity to cephems and penicillin, and are critical to high level of resistance. Two of these substitutions (in positions 311 and 483) were recently found in another highly resistant strain isolated in Australia [55].

In the laboratory, when a strain harboring a mosaic PBP2 (sequence Q8RR30) was further submitted to selection on cefpodoxime, an additional substitution G482S, which is adjacent to position 483 discussed above, was selected that provides very high resistance to cephalosporins [39]. This substitution has not been found in clinical isolates yet, but the experiment demonstrates the potential for further increase in cephalosporin resistance.

Based on sequence comparison and correlation with cephalosporin resistance, other substitutions have been proposed to contribute, without experimental verification so far, such as substitutions in helix α11 that is lining strand β4 G542S and P551S/L [56]. These positions could potentially also affect the nearby β3–β4 loop.

In susceptible strains, the meningococcal PBP2 sequence is 99% identical to that from *N. gonorrhoeae*. It is therefore unsurprising that identical substitutions and mechanisms for reducing the acylation efficiency are observed in meningococci, possibly arising from similar recombination events [19,20], or even direct horizontal transfer between the two pathogens, as they have been found to occasionally share the same niche [9]. Sequence comparisons led to the proposition of substitutions I515V, H541N and I566V, in addition to F504L and A510V discussed above, as important for resistance to penicillin and amoxicillin [19]. The role of these substitutions awaits experimental testing.

Until recently, meningococcal isolates with reduced susceptibility to penicillin G remained susceptible to third-generation cephalosporins (cefotaxime and ceftriaxone) and their minimal inhibitory concentration did not differ significantly between penicillin susceptible and penicillin resistant isolates [57]. We have lately identified a new altered *penA* allele (*penA*327) that has been increasingly detected since 2012 and that harbors not only the four altered positions proposed above (F504L, A510V, I515V and H541N), but also the I312M, V316T, N512Y and G545S substitutions. These isolates were associated not only with penicillin intermediate resistance phenotype but also with 10 fold-increased minimal inhibitory concentration to third-generation cephalosporins (our unpublished data). The *penA327* allele was responsible for this phenotype as it conferred this new phenotype when transformed into a cephalosporins susceptible isolate. All these isolates belonged to the same meningococcal genotype (the clonal complex ST-11) suggesting a clonal expansion. PBP2 encoded by allele *penA327* is identical to PBP2 XXXIV that was found in *N. gonorrhoeae* to be associated with increased resistance to third-generation cephalosporins. Interestingly, the addition of the A501P alteration in this sequence further increased the high-level expanded-spectrum cephalosporin resistance in gonococci [38]. The same point mutation may be expected to be selected in meningococci.

Taking advantage of the number of published PBP2 sequences associated with minimal inhibitory concentrations for three β-lactams (penicillin, cefixime and ceftriaxone) as well as the available structure, a proteochemometric computational model has been developed [58]. The substitutions important for the resistance identified in this way are in good agreement with those established experimentally. Most importantly, the computational model may prove useful to predict the efficacy of novel β-lactams and possible future mutations.

Thus, it appears that *penA* alleles that confer penicillin resistance have arisen both from the recruitment of sequence blocks from naturally resistant species, such as *N. flavescens*, and point mutations such as a codon insertion or substitution. When, how often, and in which species these recombination and mutation events have occurred are difficult questions. As commensal *Neisseria* species readily exchange genetic material, the *penA* alleles conferring resistance may be considered to form a common gene pool, which is shared by several species [59,60,61]. This concept has been applied previously to *S. pneumoniae*, *Streptococcus mitis* and *Streptococcus oralis,* also in the context of β-lactam resistance [62].

Early studies hinted at the possibility that PBP1, the class A PBP, also had decreased reactivity for penicillin in gonococci [16], but subsequent studies failed to uncover mosaicity in the *ponA* gene encoding PBP1. An allele of *ponA* encoding PBP1 with the single substitution L421P was found to contribute to the high resistance of some *N. gonorrhoeae* strains [63]. This substitution is 40 residues N-terminal to the catalytic S461. The L421P substitution was shown in vitro to diminish the acylation efficiency of PBP1 by various β-lactams about 4-fold [63]. It is intriguing that no mosaicity was found in *ponA*, since three *pbp* genes are mosaic in β-lactam resistant streptococci, for instance [42]. It is possible that to give rise to mosaicity, genes encoding PBPs with an intrinsic low reactivity for β-lactams must preexist in the compatible gene pool available for transfer and homologous recombination.

## Figures and Tables

**Figure 1:**
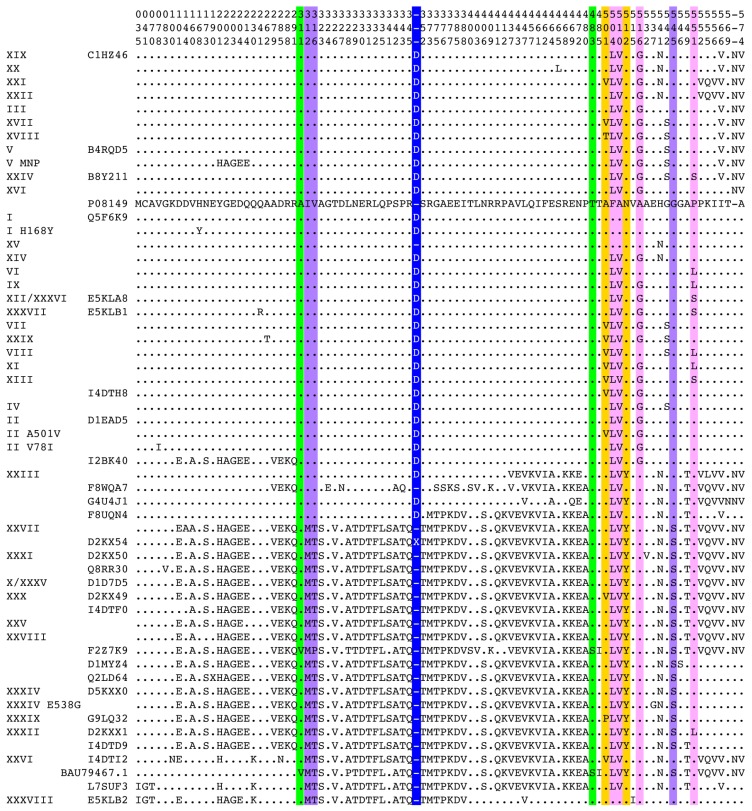
Alignment of PBP2 sequences from *N*. *gonorrhoeae* (aligned and clustered with CLUSTALW). The Roman numeral nomenclature and the Uniprot accession number are given on the left. Only positions where at least one sequence differs from the reference sequence from the susceptible strain LM306 [31] (Uniprot accession number #P08149) are shown. Substitutions or insertion shown experimentally to contribute to resistance are highlighted in color: blue [32,33]; pink [34,35]; violet [22]; yellow [34,36,37,38]; and green [39]. Yellow substitutions are specific to resistance to third generation cephalosporins. Green substitutions are responsible for high-level resistance, including to third-generation cephalosporins. This representation allows to clearly distinguish two types of sequences from resistant strains, either with the D346a insertion and few substitution clustered in the C-terminal part, or without the D346a insertion with over 50 substitutions spanning the whole protein.

**Figure 2:**
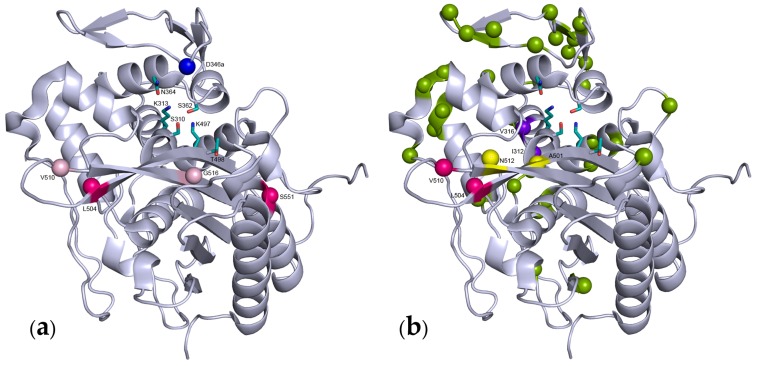
Ribbon representation of the transpeptidase domain of two types of *N. gonorrhoeae* PBP2 from resistant strains. The active site residues are shown as sticks, the substituted or inserted positions are shown by colored spheres. (**a**) Sequence XII, the D346a insertion is shown in blue; the F504L, A510V, A516G and P551S substitutions are in dull pink or magenta, to emphasize the importance of the latter substitutions in the resistance; (**b**) Distribution of the substitutions found in sequence XXXIX shown as colored spheres. Positions demonstrated as important for resistance in different studies are shown in violet [22], magenta [34] and yellow [36]. Yellow substitutions are specific of resistance to third generation cephalosporins.

**Figure 3:**
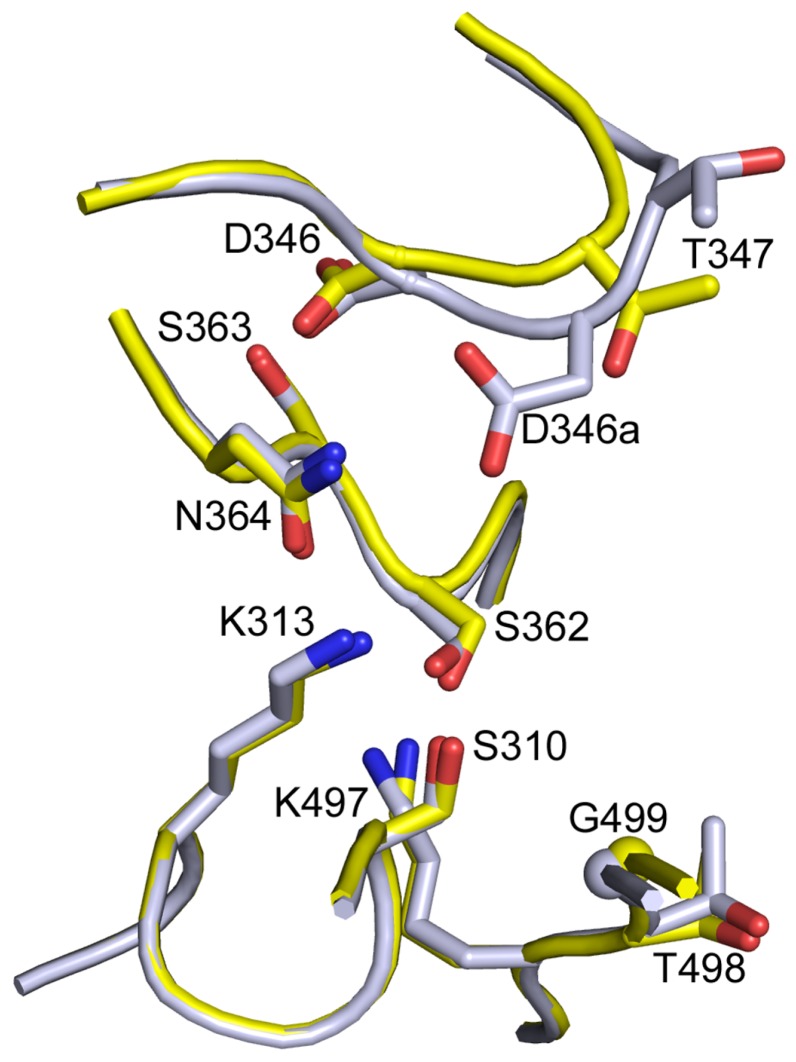
Superimposition of the structures of the active site of *N. gonorrhoeae* PBP2 with sequence XII (grey) and sequence P08149 (yellow), which are from resistant and susceptible strains, respectively. The additional D346a side chain protrudes inside the active site, towards the reactive serine 310.
